# A single high-fat meal provokes pathological erythrocyte remodeling and increases myeloperoxidase levels: implications for acute coronary syndrome

**DOI:** 10.1038/s41374-018-0038-3

**Published:** 2018-03-23

**Authors:** Tyler W Benson, Neal L Weintraub, Ha Won Kim, Nichole Seigler, Sanjiv Kumar, Jonathan Pye, Tetsuo Horimatsu, Rod Pellenberg, David W Stepp, Rudolf Lucas, Vladimir Y Bogdanov, Sheldon E Litwin, Julia E Brittain, Ryan A Harris

**Affiliations:** 1 Vascular Biology Center, Medical College of Georgia, Augusta University, Augusta, GA 30912, USA; 2 Georgia Prevention Institute, Medical College of Georgia, Augusta University, Augusta, GA 30912, USA; 3 Department of Pharmacology and Toxicology, Medical College of Georgia, Augusta University, Augusta, GA 30912, USA; 4 Department of Internal Medicine, University of Cincinnati College of Medicine, Cincinnati, OH, USA; 5 Cardiology Division, Medical University of South Carolina, Charleston, SC, USA

## Abstract

High-fat meal (HFM) consumption can produce acute lipemia and trigger myocardial infarction in patients with atherosclerosis, but the mechanisms are poorly understood. Erythrocytes (red blood cells, RBCs) intimately interact with inflammatory cells and blood vessels and play a complex role in regulating vascular function. Chronic high-fat feeding in mice induces pathological RBC remodeling, suggesting a novel link between HFM, RBCs, and vascular dysfunction. However, whether acute HFM can induce RBC remodeling in humans is unknown. Ten healthy individuals were subjected to biochemical testing and assessment of endothelial-dependent flow-mediated dilation (FMD) before and after a single HFM or iso-caloric meal (ICM). Following the HFM, triglyceride, cholesterol, and free fatty acid levels were all significantly increased, in conjunction with impaired post-prandial FMD. Additionally, peripheral blood smears demonstrated microcytes, remodeled RBCs, and fatty monocytes. Increased intracellular ROS and nitration of protein band 3 was detected in RBCs following the HFM. The HFM elevated plasma and RBC-bound myeloperoxidase (MPO), which was associated with impaired FMD and oxidation of HDL. Monocytic cells exposed to lipid in vitro released MPO, while porcine coronary arteries exposed to fatty acids ex vivo took up MPO. We demonstrate in humans that a single HFM induces pathological RBC remodeling and concurrently elevates MPO, which can potentially enter the blood vessel wall to trigger oxidative stress and destabilize vulnerable plaques. These novel findings may have implications for the short-term risk of HFM consumption and alimentary lipemia in patients with atherosclerosis.

## Introduction

Chronic consumption of meals rich in calories and saturated fats promotes obesity and adversely impacts cardiovascular health by initiating a cascade of inflammation, insulin resistance, oxidative stress, dyslipidemia, endothelial dys-function, and atherosclerosis [1]. In humans, a single high-fat meal (HFM) has been shown to increase circulating lipids, inflammatory mediators, and free radical production while activating inflammatory cells and provoking endothelial dysfunction [2]. Indeed, consuming an HFM sufficient to promote lipemia has been reported to trigger acute coronary syndromes in patients with established atherosclerosis, likely in part through activation of inflammatory cells such as monocytes and macrophages [3].

Studies examining the impact of an HFM have focused heavily on inflammatory cells. The erythrocyte (red blood cell, RBC), however, is the most abundant cell in the circulation; on average, there are 700 RBCs for every circulating leukocyte. Although RBCs are widely recognized for their vital role in transporting and delivering oxygen to the tissues, they play a much broader role in cardiovascular pathophysiology. For example, RBCs regulate vascular function via releasing adenosine triphosphate (ATP) and modulating nitric oxide (NO)-dependent vasorelaxation [4]. In addition, RBCs regulate the levels of key circulating chemokines, such as monocyte chemoattractant protein (MCP)-1, through binding to the Duffy antigen receptor for chemokines (DARC), a non-signaling receptor that is thought to function primarily as a chemokine reservoir [5]. Moreover, the RBC membrane is host to myeloperoxidase (MPO) whose binding induces vascular remodeling and stiffness, and likely contributes to endothelial dysfunction [6, 7]. Notably, MPO is a potent inducer of oxidative stress via production of hypochlorous acid, and plasma levels of MPO have been positively linked to risk of acute coronary syndromes in humans [8]. However, the mechanisms that regulate circulating and RBC-bound MPO are poorly understood.

Structurally, RBCs are composed of biconcave membranes that contain large amounts of cholesterol and avidly bind to lipoproteins, resulting in lipid transfer and membrane remodeling [9]. Moreover, RBC cholesterol levels are strongly and independently predictive of acute coronary syndromes in patients with angina [10]. We reported that chronic high-fat feeding in mice induced structural, biochemical, and functional alterations in RBCs, suggesting a role for pathologically “remodeled” RBC that may accentuate inflammation and vascular dysfunction [11]. In the current study, we sought to examine the effects of a single HFM, in comparison to a low-fat iso-caloric meal (ICM), on RBCs in a cohort of young healthy adults.

## Materials and methods

### Participant recruitment

Ten apparently healthy, physically active male participants with no past medical history and taking no prescription medications were recruited for the study, which was approved by the Institutional Review Board of Augusta University. After obtaining informed consent, a comprehensive assessment of cardiovascular disease (CVD) risk factors, vital signs, and body composition was conducted and a cardiopulmonary exercise test was performed. The protocol consisted of two testing visits separated by a minimum of 7 days. For each visit, all participants were instructed to report to the Laboratory of Integrative Vascular and Exercise Physiology at Augusta University following an overnight fast having abstained from caffeine or strenuous physical activity for 24 h and vitamin supplementation for 72 h prior to investigation.

### Experimental design

After arrival to the test center, pre-meal metabolic parameters and blood samples were collected, and flow-mediated dilation (FMD) testing was performed. After baseline assessments were performed and blood samples were collected, all participants consumed either an HFM in the form of a milkshake (1 g/kg fat, 0.5 g/kg carbohydrate, and 0.15 g/kg protein for a total of 11.6 Kcals/kg of body weight) or a low-fat ICM (0.04 g/kg fat, 2.54 g/kg carbohydrate, and 0.28 g/kg protein for a total of 11.6 Kcals/kg of body weight). Four hours post-meal consumption, metabolic parameters, blood samples, and FMD data were collected again.

### Flow-mediated dilation

Endothelial function was assessed using the brachial artery FMD test and performed according to the most recent methodological guidelines [12]. In brief, subjects rested in a supine position for ~20 min prior to baseline FMD measurements. A 12 MHz linear transducer connected to a LOGIQ 7 ultrasound imagining device (GE Healthcare, UK) was used to asses FMD. To verify that measurements were taken during the end-diastolic portion of the cardiac cycle, ECG gating (Accusync 72, USA; GE Medical Systems, China) was employed. The occlusion cuff was rapidly inflated to 250 mm Hg for a duration of 5 min. Post-occlusion measurements were initiated 30 s prior to cuff deflation and recorded for at least 2.5 min. Images were acquired using Vascular Imager software (version 6.0.3, Medical Imaging Application, USA) and arterial diameter was measured using offline edge detection software (Brachial Analyzer for Research Version 5.7.0, Medical Imaging Applications, USA). Pre-meal FMD measurements were initiated upon the subject’s arrival to the laboratory, and post-meal measurements were initiated 4 h after consumption of either the HFM or the ICM. After normalization to VO_2_ max and shear rate (AUC), ΔFMD was calculated by subtracting pre-meal FMD from meal FMD.

### Blood sampling

Venus blood samples were collected from all participants before (pre) and following (post) consumption of the HFM and ICM as previously described [13]. Briefly, blood was drawn into sodium citrate, lithium heparin, and EDTA tubes for individual biomarker testing. EDTA-collected blood was assayed for chemokines (described below) or separated by centrifugation at 3000 rpm for 10 min; plasma was removed, aliquoted, and stored at −80 °C for future analysis of lipids, glucose levels, and free fatty acids. The washed and packed RBCs were aliquoted and stored for future protein analysis. Lithium-heparin-collected blood was assayed for intracellular reactive oxygen species (ROS) via flow cytometry. For preparation of blood smears, blood was collected into sodium citrate tubes, and 3 μl of whole blood was then smeared onto a slide for analysis. Mean corpuscular volume (MCV) was estimated from hematocrit and concentration of RBCs. Estimated results were verified via blood smear.

### Erythrocyte sedimentation rate (ESR)

Blood drawn into a sodium citrate tube was separated by centrifugation at 600 RCF for 10 min. ESR was then measured following a standard protocol using an EZ-Rate pipette kit (Globe Scientific Inc.). ESR (mm/h) was measured in RBC from both the HFM and ICM pre-meal and post-meal blood draws, and ΔESR was calculated by subtracting the pre-meal value from the post-meal value.

### Blood smear analyses

To examine the effects of an HFM on RBC morphology, whole blood smears were prepared for each participant at both pre and post HFM and ICM. Whole blood was smeared onto glass cover slips using an automated device to ensure even and consistent coverage for counting morphology across samples and conditions. The smear was then stained using modified Wright–Giemsa staining. RBC morphology was scored by the blinded investigator according to Bessis et al., and is presented as raw score without weighted group average [14].

### Milliplex bead array

Blood collected in EDTA tubes was split into two equal volume aliquots; one was treated with 50 U/ml of heparin to liberate chemokines from the RBCs [7] while the other aliquot was left untreated to measure chemokines circulating in plasma. After heparin treatment, blood was separated and plasma was aliquoted and snap frozen using liquid nitrogen. Using a Milliplex MAP Human Chemokine Magnetic Bead Panel (EMD Millipore), plasma was assayed for MCP-1, IL-8, and IP-10 levels at both pre and post HFM and ICM.

### Flow cytometry

Measurement of intracellular ROS was measured as previously described [11]. Briefly, packed RBCs were washed and treated with 5-(and-6)-chloromethyl-2′,7′-dichlorodihydrofluorescein diacetate, acetyl ester (CM-H2DCFDA, Invitrogen), then passed through an Acuri C6 flow cytometer. Quantification and comparison was performed using the mean fluorescent value. Treatment with hydrogen peroxide (H_2_O_2_) was used as a positive control.

### RBC lysis and western blot for nitrotyrosine

Protein lysates for quantification of nitrotyrosine levels in the RBC membrane were prepared as described previously [13]. In brief, packed and washed RBCs were thawed and lysed for 20 min in ice-cold lysis buffer containing 1% Triton X-100, 50 mM HEPES (pH 7.4), 137 mM NaF, and 1× protease inhibitor cocktail set III (Calbiochem-Novabichem Corp.). After an overnight incubation at 4 °C of the Triton X-100 soluble fraction with nitrotyrosine antibody at 2 μg/ml final concentration, samples where incubated with protein G sepharose beads. After washing immunoprecipitated proteins in lysis buffer three times, concentrated samples were loaded onto a 4−20% SDS-PAGE gel, transferred onto a PVDF membrane (Millipore) and blotted with anti-Band-3 antibody (Abcam).

### Plasma MPO activity

**Fig. 1:**
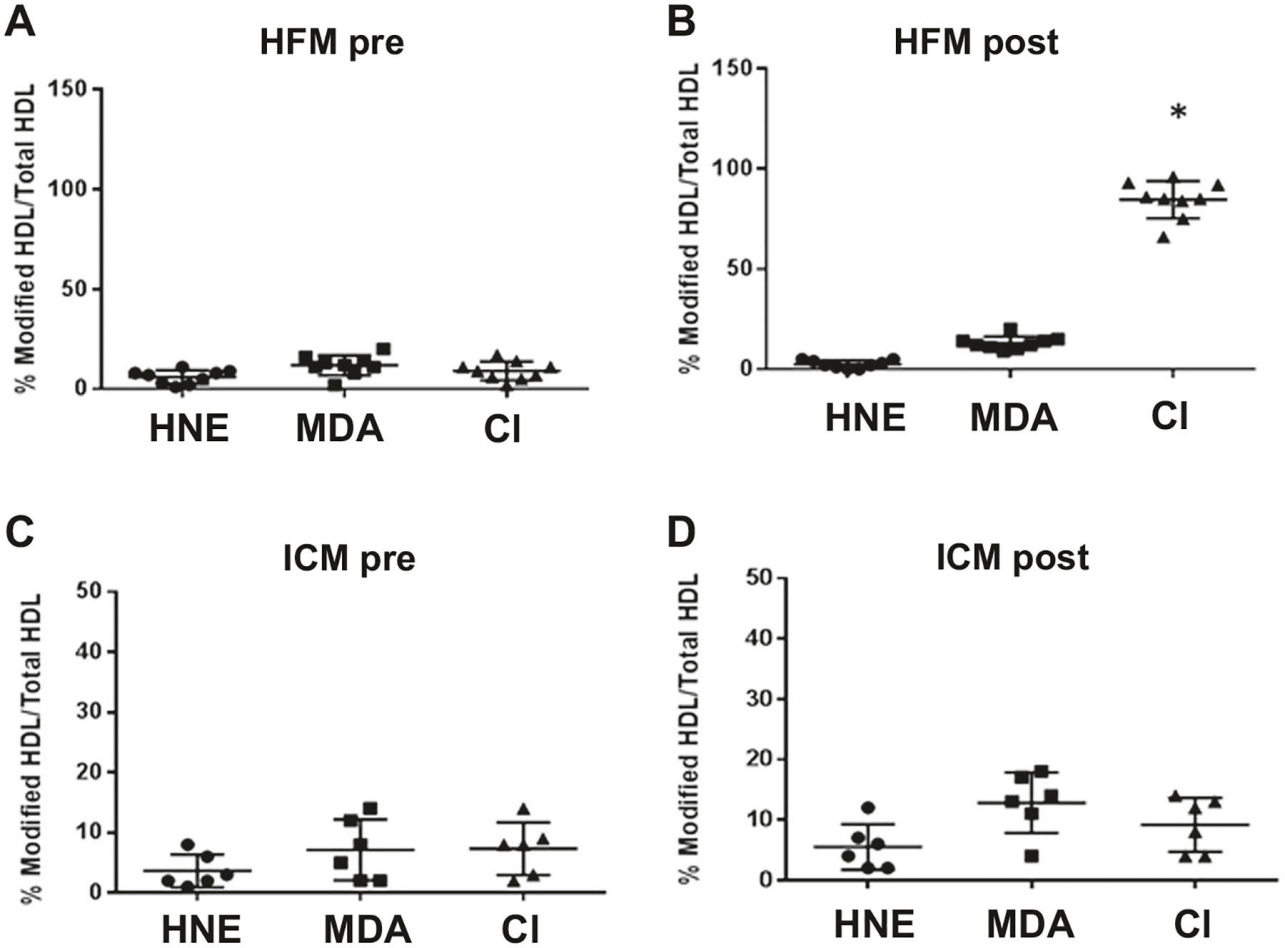
Quantification of oxidized HDL modifications before and after the HFM and ICM. HDL was interrogated in EDTA anticoagulated plasma using ELISA with IgG raised against MPO-oxidized HDL (Cl-HDL). Other modified sites (HNE and MDA) were also detected by ELISA (Generon). **a** High-fat pre, **b** High-fat post, **c** ICM pre, **d** ICM post. *Significant from HFM pre and ICM (*n* = 10 HFM, *n* = 6 ICM)

MPO activity was assayed using a colorimetric assay kit (ABCAM) as per the manufacturer’s specifications. To reduce lipemic interference in the post-HFM samples, and/or any interference of heparin (Fig. 1b), all samples were diluted 1000-fold in the kit provided buffer and assayed in triplicate. One unit of MPO activity was defined as is the amount of MPO to consume 1.0 μmol of substrate per minute at 25 °C and was normalized to plasma protein levels.

### Determination of Cl-HDL

MPO-induced modification of HDL was quantified using ELISA in which plasma HDL was captured using mouse anti-human HDL IgG (ProGen), and chlorotyrosine content was labeled using rabbit anti-chlorotyrosine (HyCult), and quantified with donkey anti-rabbit conjugated to horse-radish peroxidase (Abcam).

### Monocytic cell studies

THP-1 monocytic cells (5 × 10^5^/ml) were incubated for 4 h with 0.5% (volume/volume) triolein in complete media. The supernatant was collected, allowed to separate into aqueous and lipid phases, and the aqueous phase collected and assayed for TNF-α and IL-8 via ELISA (R&D) and MPO activity (Abcam). For fatty acid studies, the same number of cells were incubated for 4 h with 50 or 500 μM of oleic acid conjugated to albumin. Albumin was used as the control and MPO activity was determined as described above.

### Neutrophilic cell studies

HL60 cells (5 × 10^5^/ml) were incubated for 4 h with either 50 or 500 μM oleic acid conjugated to albumin. Albumin was used as the control and MPO activity was determined as described above. NB4 cells were induced to differentiate into mature granulocytes by exposure to 1 μM all trans retinoic acid (ATRA) for 72 h. Differentiation was verified via flow cytometry to detect expression of CD 11c with an anti-CD11c antibody conjugated directly to phycoerythrin. At 72 h, 83% of the cells were positive for antigen. The cells were washed and re-suspended in complete media at a concentration of 5 × 10^5^/ml and exposed to oleic acid as described for the HL60 cell line. Both cell lines were treated with 10 nM A23187 for 15 min as a positive control.

### MPO uptake by porcine coronary arteries in vitro

To model the impact of an acute HFM on MPO uptake into coronary arteries, porcine hearts were obtained from a local abattoir and immediately placed in PBS for dissection. The epicardial coronary arteries were carefully isolated and adventitial fat was removed. Vessels were cut into ~5 mm rings and pre-incubated in 200 μl of DMEM (0.1% FBS, without phenol red) in 96-well plates at 37 °C for 30 min. The rings were then exposed to bovine serum albumin-conjugated sodium palmitate or oleic acid (0–500 μM, Sigma-Aldrich) for 0–4 h, and then incubated with vehicle or purified human MPO (100 nM) for 2 h. Vessels were washed, homogenized, and MPO activity was measured by MPO detection kit (Fluoro MPO, Cell Technology) and normalized by mg of protein.

### Statistical analysis

Data are presented as mean ± SD unless otherwise noted. Significance was set at *p* < 0.05. The Shapiro–Wilk test was used to analyze the normality of the measurement distribution. Those satisfying normalcy are presented as mean ± SE. Those which fail are presented as median with interquartile range. Repeated-measures ANCOVA analyses were used to evaluate the effects of the meal (HFM vs ICM) over time (pre vs post). When a significant interaction was identified, simple main effects were performed to identify where the differences exist. The change in each outcome is expressed as delta (Δ) and calculated as the post to pre difference for each meal. Paired samples *t*-tests were used to compare Δ’s between meal. Multiple hypothesis testing significance was treated as nominal. Regression analyses were conducted using Spearman’s method.

## Results

### Participant characteristics and biochemistry

Participant characteristics are listed in Table 1. All participants were apparently healthy, non-smoking, non-obese, physically fit young adult men who completed both the HFM and ICM protocols. Biochemical parameters before and after the meals are presented in Table 2. Consistent with prior publications [15], lipemia was present 4 h following ingestion of the HFM, but not the ICM (not shown), and total cholesterol (*p* = 0.01), triglycerides (*p* = 0.01), and plasma free fatty acids (*p* = 0.003) were all significantly increased post HFM. In contrast, the ICM had no effect on these parameters. Additionally, high-density lipoprotein (HDL), low-density lipoprotein (LDL), and glucose levels were similar after both the HFM and ICM.

### Vascular endothelial function

Supplemental Figure S1 illustrates a significant meal by time interaction (*p* = 0.046) for flow-mediated dilation (FMD) when controlling for BMI. Specifically, a significant (*p* = 0.005) decrease in FMD was observed following the HFM, whereas no change (*p* = 0.837) was observed following the ICM. Additional parameters of the FMD test for pre and post meal are presented in Table 3. No differences (all *p* > 0.05) in baseline diameter, peak diameter, or absolute change in diameter were observed within or between meals. Shear rate was also similar (*p* = 0.790) pre and post within and between each meal. Consistent with the FMD findings, a significant (*p* = 0.016) meal by time interaction was observed for FMD normalized for shear rate when controlling for BMI. In addition, the pre to post change in concentrations of sVCAM, an inflammatory marker that correlates with the extent of atherosclerosis [16], was unaffected by consumption of either the HFM (Δ 3.12 ± 0.99) or the ICM (Δ −6.96 ± 2.2).

### Erythrocyte sedimentation rate and RBC morphology

**Fig. 2:**
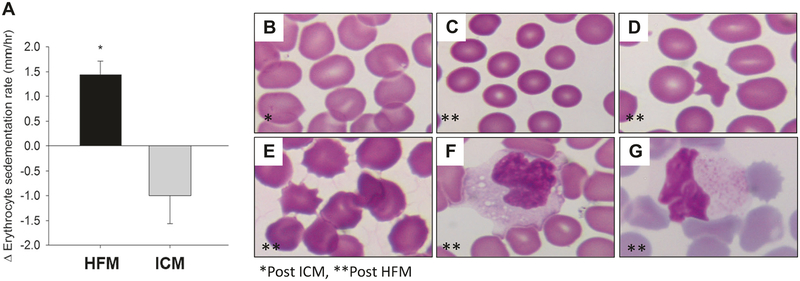
Changes in erythrocyte morphology and blood monocytes following HFM. **a** Effects of HFM and ICM on changes in erythrocyte sedimentation rate. Effects of ICM (**b**) and HFM (**c–e**) on RBC morphology. Note the appearance of microcytosis (**c**), acanthocytosis (**d**), and echinocytosis (**e**) following HFM. Foamy monocytes (**f**) and lipid-laden monocytes (**g**) post HFM. **b–e** ×400 magnification, **f–g** ×1000

The change in erythrocyte sedimentation rate (ESR) was measured as a general marker of inflammation pre and post the HFM and ICM. Figure 2a illustrates a significant increase in ESR following the HFM, whereas no change was observed in ESR following the ICM. Compared to post ICM (Fig. 2b), RBC morphology was noticeably altered following the HFM. Specifically, microcytosis (Fig. 2c) was observed in the post-HFM samples. Furthermore, there was a significant increase in the number of acanthocytes (Fig. 2d) and echinocytes (Fig. 2e) post HFM. The blinded investigator scored change in RBC morphology is presented in Table 4. Mean corpuscular volume (MCV), estimated as hematocrit divided by RBC concentration (MCV_e_), was significantly (*p* = 0.015) reduced post HFM compared to the ICM (Table 4). In addition to the changes in RBC morphology, we detected foamy monocytes (Fig. 2f) and lipid-laden monocytes (Fig. 2f) in the circulation post HFM.

HFMs have been demonstrated to transiently increase circulating chemokine levels in humans [17]. Moreover, chronic high-fat feeding in mice promoted an increase in chemokine binding to RBC DARC [11]. To investigate whether a single HFM could increase chemokines in the circulation and/or bound to RBC in our subjects, we measured MCP-1, interleukin (IL)-8, and interferon gamma-inducible protein (IP)-10 using a multiplex bead array after treating blood with or without heparin to liberate chemokines bound to RBC DARC [11]. None of the levels of these cytokines were increased by either the HFM or the ICM. Although treatment with heparin increased MCP-1, indicative of liberation from DARC, the amount released was unaffected by consumption of either the HFM or ICM (Supplemental Figure S2). Heparin treatment was not effective at liberating either IL-8 or IP-10 from RBC in our study.

### Intracellular RBC reactive oxygen species

**Fig. 3:**
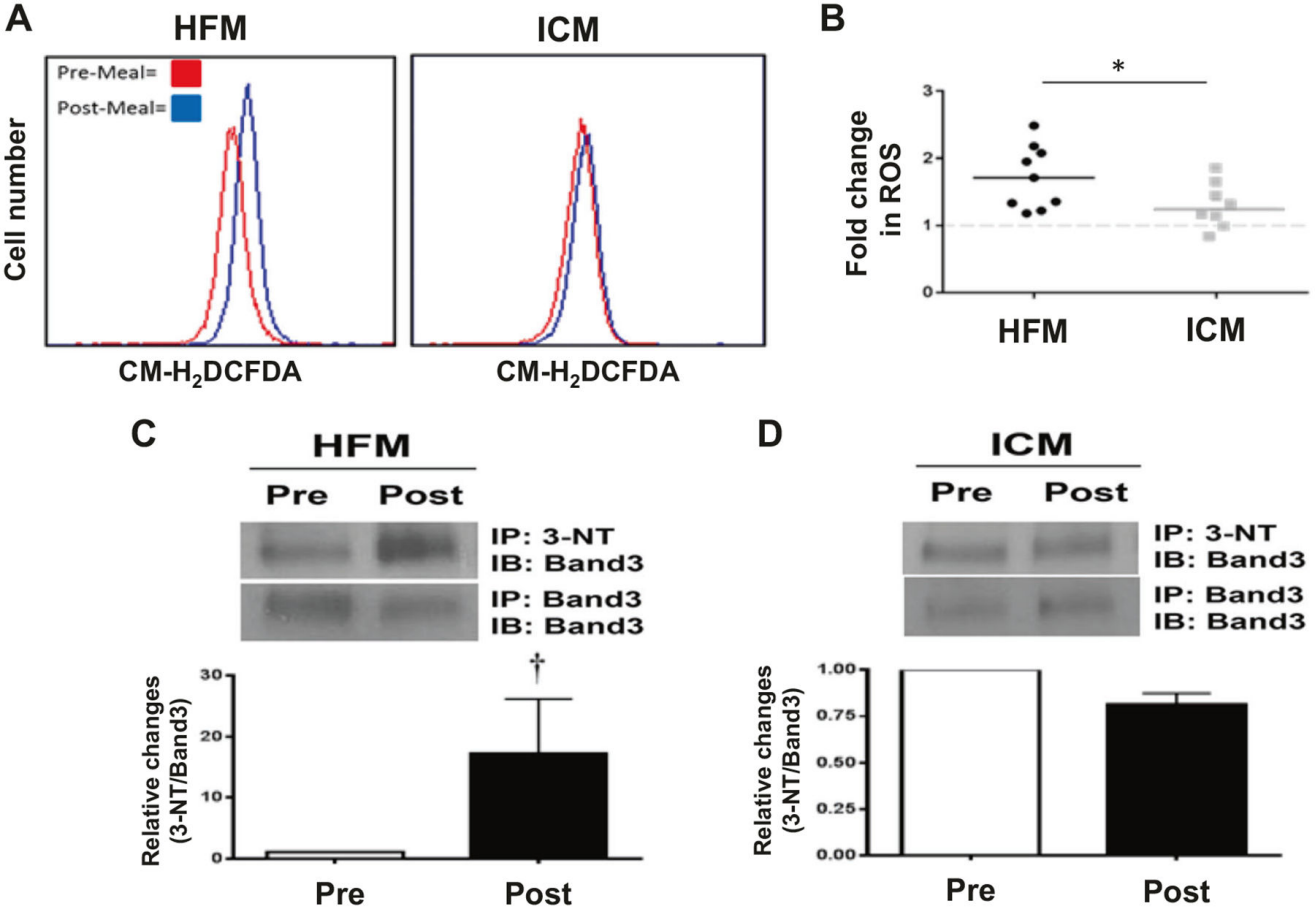
Effects of HFM and ICM on levels of ROS and oxidative stress in RBCs. a Intracellular ROS were detected in washed RBCs loaded with the probe CM-H_2_DCFDA and subjected to flow cytometry. Representative histograms show **a** positive shift in fluorescence 4 h post HFM (left), while there was no significant shift following ICM (right). **b** Quantification of the fold change in fluorescence post meal. Each dot represents an individual subject value, and the horizontal lines denote the mean values (*n* = 10 HFM, *n* = 8 ICM). Tyrosine nitration of band 3 isolated from RBC membranes was increased in HFM group (**c**), while no change was observed in ICM group (**d**). *Significant from ICM. ^†^Significant from Pre-meal (*n* = 6)

The effects of the HFM on intracellular RBC reactive oxygen species (ROS) were determined using a fluorescent probe, CM-H_2_DCFDA. Representative histograms produced from RBC pre and post HFM demonstrate a positive post-prandial shift in fluorescence following HFM, indicative of increased intracellular ROS in the RBCs (Fig. 3a, left). In contrast, no significant increase in ROS was detected following ICM (Fig. 3a, right). Figure 3b illustrates the significantly greater (*p* < 0.05) fold change in ROS following the HFM compared to the ICM. To determine whether the increase in ROS induced by the HFM was sufficient to induce oxidative damage to the RBCs, proteins were harvested from the RBC membranes pre and post HFM or ICM and assayed by western blotting. Notably, significant increases in tyrosine nitration (3-NT) of band 3 (anion exchanger 1) were detected following the HFM (*p* = 0.008), whereas no change was observed following the ICM (Fig. 3c, d).

### Plasma and RBC-bound MPO

**Fig. 4:**
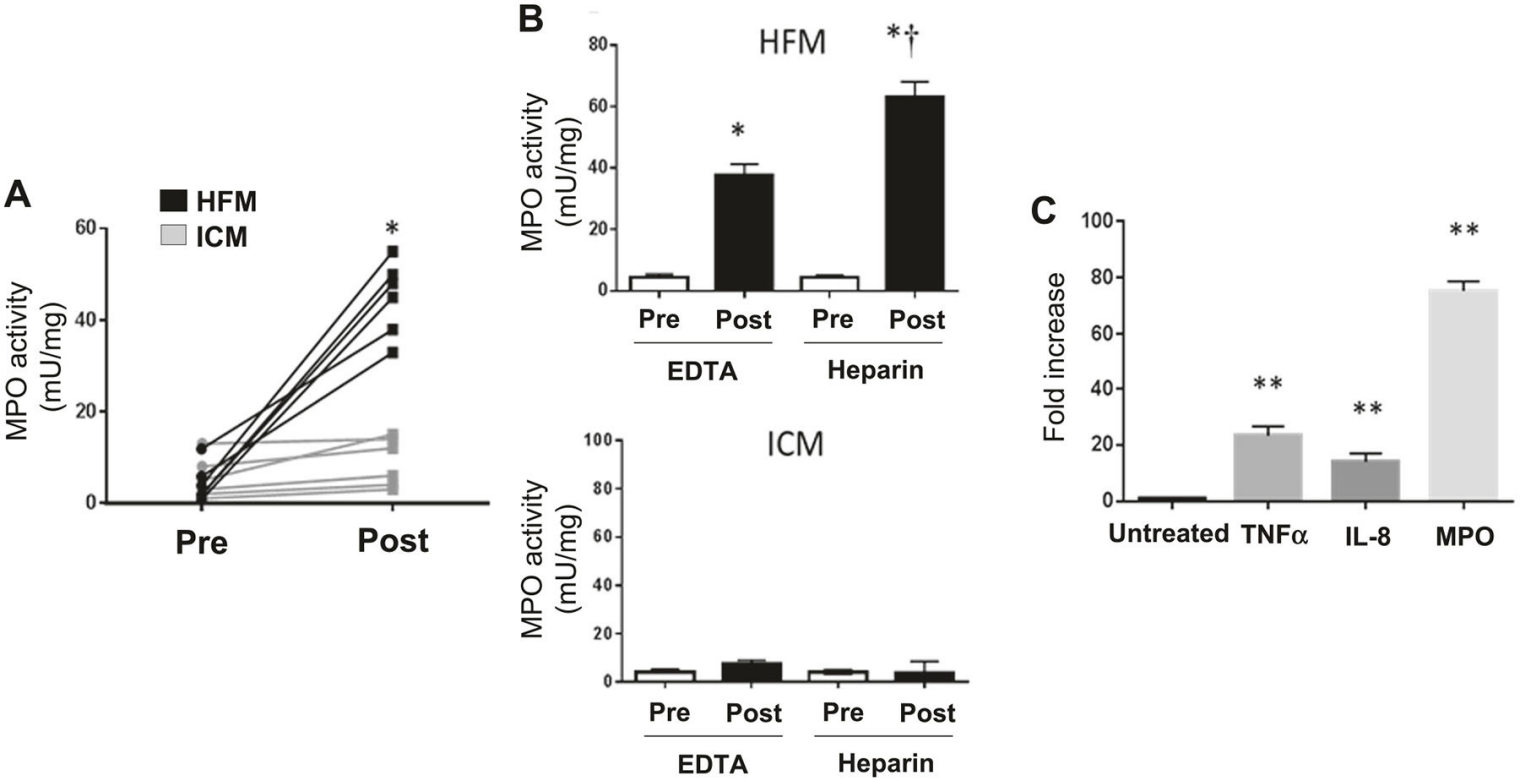
Changes in MPO activity following the HFM and ICM and monocitic exposure to neutral fat. **a** Individual plasma MPO activity levels before (pre) and after (post) the HFM and ICM. **b** Pre and post plasma MPO activity following the HFM (top) and ICM (bottom) in EDTA or heparin anticoagulated samples. **c** Relative changes in MPO activity and inflammatory marker expression (ELISA) in response to THP-1 monocyte exposure to neutral fat for 4 h. *Significant from pre. **Significant from untreated. ^†^Significant from EDTA post (*n* = 6)

We next examined the activity of MPO in the plasma of participants pre and post HFM or ICM. Figure 4a illustrates the significantly (*p* = 0.002) greater elevation in MPO following the consumption of the HFM compared to the ICM. To determine whether RBC-bound MPO was also increased following the HFM, we assayed plasma for MPO activity using heparin to liberate MPO from RBCs. While there was an increase in MPO activity in the EDTA-collected samples post HFM, collection of these samples into heparin resulted in a further increase in MPO activity, indicative of liberation from RBCs (Fig. 4b). In contrast, very little MPO activity was detected in EDTA- or heparin-collected samples post ICM (Fig. 4b). Interestingly, MPO released from the RBCs upon heparin collection correlated significantly with the impairment in FMD (r = 0.63, p = 0.023), whereas the baseline collection into EDTA did not (*r* = 0.21, *p* = 0.68), consistent with a potential role for RBC-bound MPO in promoting endothelial dysfunction post-HFM consumption.

To investigate potential mechanisms that might promote an increase in circulating MPO following the HFM, monocytic THP-1 cells were either untreated or exposed to a neutral fat, triolein, for 4 h, after which MPO in the medium was assayed. Exposure to triolein resulted in significant (*p* < 0.05) MPO release when compared to the untreated cells (Fig. 4c). In addition, a significant release of the inflammatory factors tumor necrosis factor (TNF)-α and IL-8 was detected from THP-1 monocytic cells upon exposure to triolein (Fig. 4c). To determine whether oleic acid, the primary fatty acid present in the HFM, could induce a similar response, THP-1 cells were exposed to albumin-conjugated oleic acid for 4 h and MPO activity was measured. Oleic acid treatment dose dependently induced the release of active MPO as compared to albumin treatment alone (Supplemental Figure S3).

The primary MPO releasing cells in the blood are the granulocytes, with neutrophils being the most prevalent; thus, we also examined whether free fatty acids can induce MPO release from neutrophilic cells. Albumin-conjugated oleic acid (50 or 500 μM for 4 h) failed to induce MPO release from either HL60 cells or ATRA-differentiated NB4 cells, whereas both cell lines mounted a brisk degranulation response to 10 nM of the calcium ionophore A23187 within 15 min of treatment (Supplemental Figure S4). These data suggest that free fatty acids exhibit specificity with regard to eliciting MPO release from the monocytic cells in blood.

**Fig. 5:**
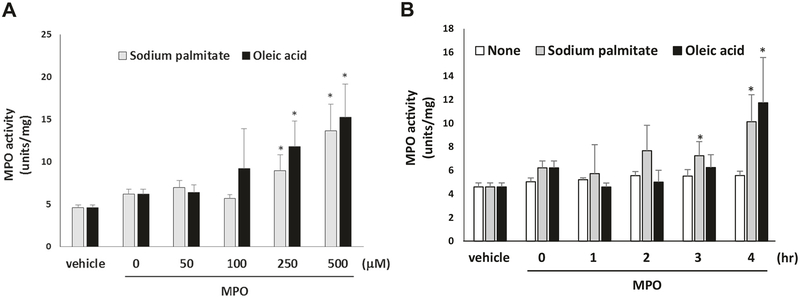
Effects of free fatty acids on MPO uptake by porcine coronary artery. Porcine coronary arterial rings were exposed to varying concentrations of **a** bovine serum albumin-conjugated sodium palmitate or oleic acid for 4 h, or to **b** 300 μM sodium palmitate or oleic acid for variable durations, followed by incubation with purified human MPO (100 nM) for 2 h. MPO activity in the arterial rings was then quanti-fied. *Significant from vehicle treatment (*n* = 3)

Given the pro-atherosclerotic potential of MPO, we investigated whether the HFM-induced increase in MPO was sufficient to promote pro-atherogenic oxidative modification of HDL. We observed that consumption of the HFM (Fig. 1b), but not the ICM (Fig. 1d), increased the levels of chlorotyrosine (Cl), a specific marker for MPO-mediated oxidative modification of HDL. In contrast, modifications of HDL linked to other oxidant mediators (hydroxynonenal; HNE, malondialdehyde; MDA) were not detected in any of the assayed samples. Finally, because the HFM was associated with concurrent increases in circulating free fatty acids and MPO, we investigated whether incubation of coronary arteries with fatty acids could promote vascular MPO uptake. Porcine coronary arteries were exposed to sodium palmitate, oleic acid, or vehicle and incubated with MPO. Afterward, the arteries were homogenized and MPO activity was assayed. Notably, incubation with either palmitic or oleic acid, the two most abundant fatty acids contained in bovine milk fat [18] induced a significant (*p* < 0.05) increase in MPO uptake into porcine coronary arteries in a dose- (Fig. 5a) and time-dependent manner (Fig. 5b).

## Discussion

RBCs intimately interact with blood vessels and are increasingly recognized for their complex role in regulating vascular function and cardiovascular-related disease. Here, we demonstrate for the first time in humans that a single HFM sufficient to induce lipemia promotes RBC remodeling, induces intracellular ROS and oxidative damage to RBC membranes, and increases circulating and RBC-bound MPO that is sufficient to promote oxidative modification of HDL. Additionally, in vitro, monocytic cells exposed to lipid release MPO, which in turn is taken up by coronary arteries in the presence of free fatty acids. These findings may have implications with regard to the mechanisms, whereby consumption of meals rich in fat have been temporally linked to the development of acute coronary syndromes in humans [3, 19].

### Erythrocyte morphology

Examination of whole blood smears (Fig. 2b–e) demonstrated a post-prandial increase in microcytes induced by the HFM. This change in morphology was likely caused by lipemia-induced osmotic forces imposed on the RBC. Interestingly, microcytes have been reported to exhibit less deformability and a tendency to clump in the micro-circulation, suggesting potentially important functional consequences with regard to blood rheology, increased RBC density, and increased peripheral resistance [20]. In addition, acanthocytes and echinocytes were also detected following consumption of the HFM in most individuals. Structural alterations in protein band 3 have been associated with increased presence of acanthocytes [21], and MPO binding has been suggested to lead to the formation of echinocytes. Thus, the increased tyrosine nitration of band 3 post HFM, combined with the increase in RBC MPO, suggest multiple hits to the RBC each of which compromise its vaso-regulatory function. Indeed, even non-specific RBC clumping or aggregation in addition to impaired deformability has been suggested to mediate increased micro-vascular flow resistance and subendocardial ischemia in patients with angina [22]. Whether the alterations in RBC morphology identified in this study might have contributed to the impaired FMD is unknown and beyond the scope of this investigation. Future studies are certainly warranted to identify if changes in erythrocyte morphology contribute to changes in vascular health.

Monocytes patrol the circulation in search of diseased or senescent RBC; for example, those infected by blood-borne pathogens such as malaria [23]. This function of monocytes is crucial for proper immune surveillance and infection control. In fact, our study detected foamy monocytes after a single HFM (Fig. 2g). We also demonstrated that ingestion of the neutral fat triolein was sufficient to induce the release of MPO, and other inflammatory factors, from monocytic cells. Furthermore, increased MPO release was detected in monocytic cells, but not in neutrophilic cells, when exposed to oleic acid, the primary fatty acid present in HFM, suggesting that monocytic cells are a major cell source for MPO release by HFM. Conceivably, RBCs that are sufficiently “remodeled” by the HFM might be mistakenly recognized by circulating monocytes as infected or damaged. Indeed, we recently reported that remodeled RBCs from mice fed a chronically high-fat diet were avidly taken up by macrophages in vitro, triggering proinflammatory gene expression and cytokine production [11]. Moreover, when injected in vivo, the remodeled RBCs were avidly taken up by the spleen, and when incubated in vitro, they provoked increased monocytic cell binding to the vascular endothelium. The presence of acanthocytes following the HFM is consistent with the homing and escape of damaged RBCs to and from the spleen. Collectively, these findings suggest that the immune system may be capable of recognizing and responding to RBCs that are remodeled by consumption of the HFM, analogous to the “molecular mimicry” induced by oxidized lipid epitopes in atherosclerosis [24].

### Role of MPO and clinical relevance

Plasma MPO levels have been linked to risk of acute coronary syndrome in several studies [8, 25, 26]. A transient increase in MPO, consequent to a single HFM, is not likely to be harmful to an otherwise healthy individual free of underlying atherosclerosis. However, uptake of MPO into a diseased coronary artery could potentially contribute to destabilization of vulnerable plaques [8]. Our ex vivo data show that MPO uptake in porcine coronary artery was augmented by treatment with free fatty acids, which have been shown to promote endothelial dysfunction [17]. MPO is known to bind to endothelial cells and accrues in the vascular matrix via endothelial transcytosis [27], but whether this process may be perturbed by fatty acids is unknown. Further in-depth studies are required to determine whether fatty acids augment vascular MPO uptake through the endothelium and/or by transcellular or paracellular pathways. Nevertheless, it is clear that the single HFM significantly increased plasma and RBC-bound MPO (Fig. 4) and promoted MPO-mediated oxidative modification of HDL (Fig. 1). These data suggest that HFM can transiently increase circulating MPO levels and potentially promote MPO uptake into the vascular wall, which in turn could contribute to oxidative stress, plaque destabilization, and acute coronary syndrome.

### Inflammatory consequence of the HFM

HFMs have been demonstrated to transiently increase circulating chemokine levels in humans [28]. Moreover, chronic high-fat feeding in mice promotes an increase in chemokine binding to RBC DARC [11]. In the present study, and in contrast to prior reports [10], a single HFM was insufficient to induce changes in pro-inflammatory chemokines or sVCAM levels in this young, non-obese cohort. The reason for this discrepancy is unclear. However, given that we only tested a single post-prandial time point (4 h) following consumption of the HFM, it is possible that a transient increase in chemokines was not detected. Indeed, monocytic cells exposed in vitro to lipid produced measurable levels of TNF-α and IL-8, consistent with inflammatory activation (Fig. 4c). However, the 4 h time point employed in the in vivo study was specifically chosen based on data from prior published studies, which demonstrated elevated chemokine levels. More likely, the single HFM did not evoke a measurable chemokine response in this cohort because all participants were young, non-obese, and exceptionally fit as evidenced by the high peak VO_2_ values (Table 1). Predictably, these subjects were highly insulin sensitive and exhibited a very low level of basal adipose tissue and systemic inflammation, so that the HFM challenge was well tolerated. In fact, published data from our team indicate an equivocal vascular response to an HFM between active and sedentary men [2]. Nonetheless, ESR, a general marker of inflammation, was increased following the HFM (Fig. 2a). The elevated ESR might have been due to increased plasma viscosity resulting from the HFM, which may in turn have been related to changes in the RBC membrane. Both inflammation and viscosity are believed to be mechanistically linked to destabilization of coronary atheromatous plaques leading to myocardial infarction [29].

### Erythrocyte-induced ROS

Impaired FMD induced by the HFM is thought to be mediated by post-prandial lipemia and elevated ROS, both of which can diminish NO bioavailability [30]. In the present study, we detected a significant increase in intracellular RBC ROS following the consumption of a single HFM. Although the source of the ROS and the specie(s) of free radicals remains to be determined, RBCs are particularly susceptible to oxidative stress due to their high content of unsaturated lipids and O_2_ [31]. Importantly, we also showed that the increased ROS post HFM was sufficient to induce oxidative RBC damage as measured by tyrosine nitration of band 3 (Fig. 4). Oxidative damage to the RBC has been implicated in the disruption of the cytoskeletal network, resulting in complexes between spectrin, oxidized hemoglobin, and band 3^29^. Disruption of the cytoskeleton has in turn been shown to impair membrane deformability and impede RBC passage through microcirculation, which may produce detrimental effects on blood flow. Increases in non-fasting circulating cholesterol/triglycerides and endothelial dysfunction have been identified as risk factors for atherosclerosis, which raises the possibility that RBC-induced ROS provoked by the HFM could also be a contributory factor [15].

Consumption of a single HFM, but not an ICM, resulted in a significant increase in circulating concentrations of total cholesterol, triglycerides, and free fatty acids (Table 2), findings that are in line with what has previously been reported [15]. In addition, the HFM resulted in a reduction in brachial artery FMD, which has been associated with increased risk of atherosclerotic cardiovascular disease [32]. Taken together, our findings may provide insight into the risk of acute coronary syndromes occurring following consumption of copious, fat-enriched meals. This may be particularly important to emphasize given the controversy surrounding the association between chronic fat consumption and cardiovascular disease, which has led some professionals to recommend relaxing restrictions on dietary fat intake [33, 34]. Unfortunately, patients might interpret such recommendations to mean that consuming fatty foods, no matter what the amount, is not harmful. Based on the present findings, the potential risk of “binge” HFM may be greater than previously recognized for patients with coronary artery disease, akin to the risk of other binge habits such as alcohol consumption.

In conclusion, we demonstrate for the first time in humans that a single HFM can induce pathological RBC remodeling and oxidative stress, in conjunction with elevations in plasma and RBC-bound MPO. Remarkably, the single HFM was sufficient to promote MPO-mediated HDL oxidation. Together, these findings shed novel insight into the mechanisms whereby consumption of heavy meals enriched in fat may promote destabilization of vulnerable plaques leading to acute myocardial infarction.

## Supplementary Material

Supplemental

## Figures and Tables

**Table 1:** Participant characteristics

Variable	
N	10
Age (years)	26 ±3
Height (cm)	184±7
Weight (kg)	83.8 ± 14.6
BMI (kg/m^2^)	24.7 ± 3.9
SBP (mm Hg)	117±12
DBP (mm Hg)	65 ±4
Body fat (%)	22.5 ± 4.2
Framingham risk score	−5.9 ± 2.7
VO_2_ peak (ml/kg/min)	56.7 ± 9.4

Values are mean ± SD

*BMI* body mass index, *SBP* systolic blood pressure, *DBP* diastolic blood pressure

**Table 2:** Blood testing following the high fat and iso-caloric meals

Variable	PreHFM	Post HFM	Pre ICM	Post ICM
Total cholesterol (mg/dl)	162±12	173 ±14^a^	148±9	146 ± 10
HDL (mg/dl)	48 ± 5	49 ± 5	47 ± 5	46 ±5
LDL (mg/dl)	100±9	86 ±12	92 ± 7	85 ±4
Triglycerides (mg/dl)	87 ±12	211 ±42^a^	83 ± 6	98 ±5
Free fatty acids μm/l)	135±15	203 ±12^a^	109 ± 17	40± 11
Glucose (mg/dl)	90 ± 3	86 ± 2	90 ± 2	85 ±6

Values are mean ± SEM

*HDL* high density lipoprotein, *LDL* low density lipoprotein

a Significant from pre HFM

**Table 3:** Parameters of flow-mediated dilation testing

Variable	Pre HFM	Post HFM	Pre ICM	Post ICM
Baseline diameter (mm)	0.382 ±0.009	0.0388 ±0.010	0.374 ±0.014	0.037 ±0.014
Peak diameter (mm)	0.409 ±0.010	0.410 ±0.010	0.040 ±0.014	0.398 ±0.013
Absolute change (mm)	0.026 ±0.004	0.022 ±0.004	0.024 ±0.003	0.025 ± 0.004
Shear rate (s-1, AUC)	35216 ±2248	34962 ± 3454	33608 ± 3246	34473 ± 2420
FMD/shear (%/S^−1^, AUC)	0.194 ±0.029	0.163 ±0.027^a^	0.0196 ±0.021	0.210 ±0.034
Time to peak dilation (s)	38 ±3	44±4	41 ± 4	45 ± 3

Values are mean ± SEM

a Significant from pre HFM

**Table 4:** Erythrocyte morphology change score following the HFM and ICM

	Erythrocyte scoring summary		
	Acanthocytes	Echinocytes	MCV_e_
Change following HFM	2+	3	74 [63.0, 80.7]
Change following ICM	ND	ND	97 [86.0, 103]

Values are presented as mean of observer scores with “+” indicating averaged value fell between 2 and 3. MCV_e_ values are shown as median with [interquartile range]

*ND* none detected
