# Supplementary material for: A single high-fat meal provokes pathological erythrocyte remodeling and increases myeloperoxidase levels: implications for acute coronary syndrome
Source: Lab Invest. Author manuscript; Available in PMC 2019 Jan 22. (PMC6342280; doi:10.1038/s41374-018-0038-3)
Supplement: Supplemental [file NIHMS1004607-supplement-Supplemental.pdf]

SUPPLEMENTARY INFORMATION

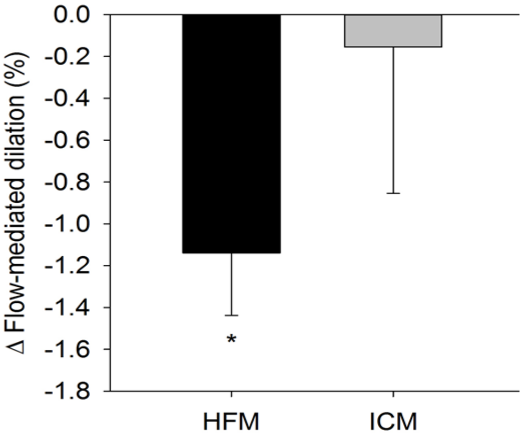

Figure S1

Figure S1. Changes in flow-mediated dilation following the HFM and ICM. *Statistically significant (p<0.05) compared to the ICM.

Figure S2

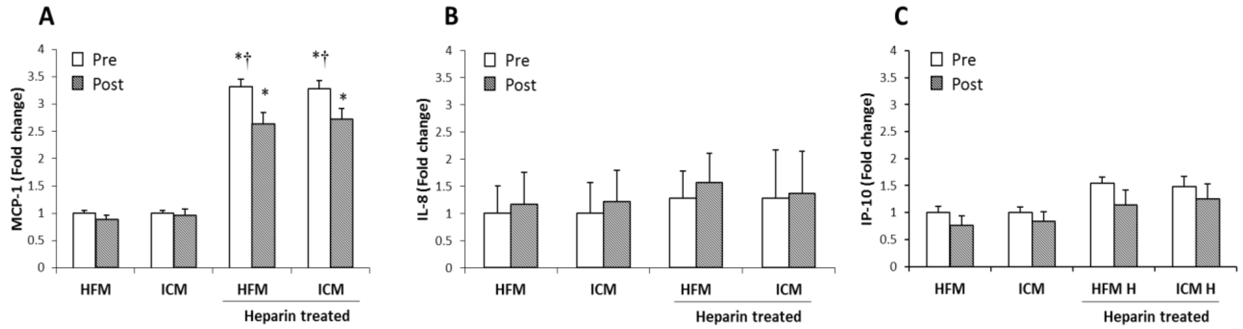

Figure S2. Effects of HFM and ICM on plasma and RBC-bound chemokine levels. Plasma MCP-1, IL-8, and IP-10 were assayed following consumption of either HFM or ICM. Heparin was used to liberate chemokines bound to DARC on RBCs. Pre refers to pre meal, and post refers to post meal. *Significant vs no heparin treatment, †Significant vs post meal.

Figure S3

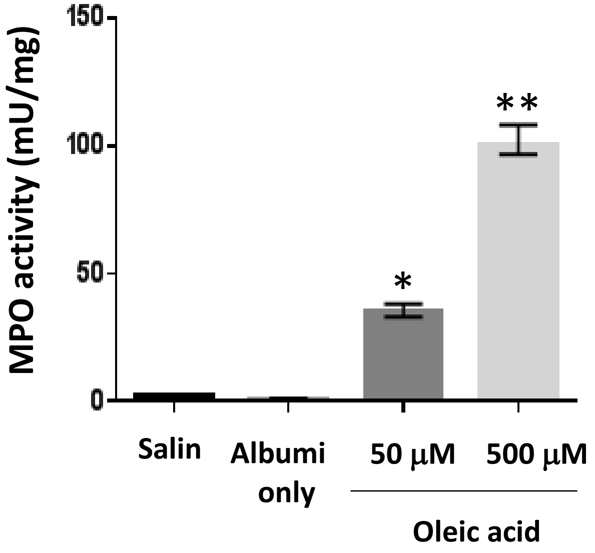

Figure S3. Effects of oleic acid on MPO release from monocytic cells. Treatment of albumin-conjugated oleic acid released active MPO from THP-1 cells in a dose-dependent manner. *Significant vs Albumin only (*p<0.05, **p<0.001, n=3).

Figure S4

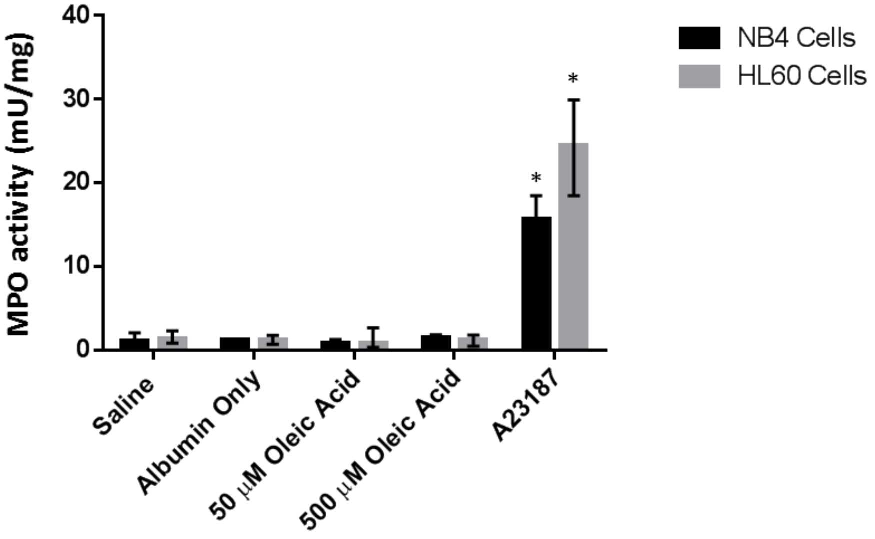

Figure S4. Effects of oleic acid on MPO release from neutrophilic cells. Treatment of albumin-conjugated oleic acid did not release active MPO in NB4 or HL60 cells. *Significant vs Albumin only (*p<0.001, n=3).
